# Consensus on the therapeutic management of rosacea – Brazilian Society of Dermatology^⋆^^⋆^

**DOI:** 10.1016/j.abd.2020.08.001

**Published:** 2020-10-10

**Authors:** Clivia Maria Moraes de Oliveira, Luiz Mauricio Costa Almeida, Renan Rangel Bonamigo, Carla Wanderley Gayoso de Lima, Ediléia Bagatin

**Affiliations:** a Dermatology Service, Universidade Federal do Pará, Belém, PA, Brazil; b Department of Dermatology, Faculdade de Ciências Médicas de Minas Gerais, Belo Horizonte, MG, Brazil; c Dermatology Service, Santa Casa de Misericórdia de Belo Horizonte, Belo Horizonte, MG, Brazil; d Dermatology Service, Hospital das Clínicas de Porto Alegre, Faculty of Medicine, Universidade Federal do Rio Grande do Sul, Porto Alegre, RS, Brazil; e Dermatology Service, Universidade Federal da Paraíba, João Pessoa, PB, Brazil; f Department of Dermatology, Escola Paulista de Medicina, Universidade Federal de São Paulo, São Paulo, SP, Brazil

**Keywords:** Consensus, Rosacea, Therapeutics

## Abstract

**Background:**

Rosacea is a chronic inflammatory disease of the skin, relatively more frequent in women over 30 with a low phototype and proven genetic predisposition. Although its etiology is unknown and possibly multifactorial, the immunological abnormality, associated with neurovascular dysregulation and triggering factors, are important elements in its pathophysiology, which lead to the main changes of inflammation, vasodilation, and angiogenesis that are responsible for the clinical manifestations. Despite the lack of cure, numerous therapeutic options are available for the different clinical presentations of the disease, with satisfactory responses.

**Objective:**

To reach a consensus, with recommendations from experts, on the therapeutic management of rosacea suitable to the Brazilian setting.

**Methods:**

The study was conducted by five specialized dermatologists from university centers, representatives of the different Brazilian regions, with experience in rosacea, who were appointed by the Brazilian Society of Dermatology. Based on the adapted DELPHI methodology, the experts contributed through an updated bibliographic review of the scientific evidence, combined with personal experiences.

**Results:**

The group of experts reached a consensus on the relevant aspects in the therapeutic management of rosacea, providing information on epidemiology, pathophysiology, triggering factors, clinical condition, classification, quality of life, and comorbidities. Consensus was defined as approval by at least 90% of the panel.

**Conclusion:**

Despite the impossibility of cure, there are several therapeutic alternatives specific to each patient that provide excellent results, with chances of total improvement and long periods of remission, promoting a positive impact on quality of life. This consensus provides detailed guidance for clinical practice and therapeutic decisions in rosacea.

## Introduction

### Epidemiology

Rosacea is a chronic inflammatory skin disease with complex etiopathogenesis, involving immunological changes and neurovascular dysregulation; there are established risk factors and clinical conditions, which can significantly compromise the quality of life of patients. It has a broad worldwide distribution (5% affected, globally), predominantly in populations of low phototypes (I and II in the Fitzpatrick classification), although studies have demonstrated that it can affect different ethnicities.^1^, ^2^, ^3^, ^4^, ^5^ A slight female predominance is observed and, although it also affects children and the elderly, the predominant age group is between 30 and 60 years.^1^, ^2^, ^6^, ^7^, ^8^

Most epidemiological reports were conducted in Europe and North America, although recent articles describe rosacea as a health problem in different regions of the world.^1^, ^2^, ^3^, ^4^, ^5^, ^9^ Recent publications certify that the disease appears to be universal and does not just affect Caucasians.^2^, ^3^, ^4^, ^5^, ^6^, ^8^, ^9^

The worldwide distribution of rosacea appears to range from 2% to 22%. Estimates in relation to prevalence in different countries are: Estonia 20%, Germany 12%, Sweden 10%, Russia 5%, United States 5%, Colombia 3%, France 3%. The rates for countries such as China, Brazil, India, and Australia are not reliably recognized.^2^, ^3^, ^4^, ^5^, ^6^, ^7^, ^8^, ^9^, ^10^

In Brazil, there are no reports on rosacea that take into account the country's population heterogeneity. A study conducted in Southern Brazil, including 62 cases and 124 controls, confirmed age between 40 and 50 years and female predominance, in addition to European ancestry, in most cases.^6^ However, there are numerous cases of patients with higher phototypes followed-up in dermatology services in different Brazilian regions, which need to be considered.

In this article, the main aspects of rosacea will be presented, in order to synthesize knowledge and indicate the best current therapeutic management.

### Pathophysiology

Although the pathogenesis of rosacea is not completely understood, it is considered a multifactorial disease.^11^ Genetic predisposition, abnormalities of the immune system, and neurovascular dysregulation – associated with triggering factors – are the main elements involved in the pathophysiology of the disease.^12^

The genetic predisposition to carry the polymorphic variant rs3733631 in the TACR3 tachykinin receptor gene, and polymorphism in the glutathione S-transferase (GST) enzyme are related to the disease.^13^, ^14^, ^15^ Chang et al. identified that genes associated with rs763035 are expressed in rosacea skin samples and identified that three class II alleles of the major histocompatibility complex (MHC), including HLA-DRB1, HLA-DQB1, and HLA-DQA1, are involved.^16^, ^17^, ^18^

Dysregulation of the innate immune response increases the secretion of antimicrobial peptides (AMP) and cytokines, *via* activation of toll-like receptor 2 (TLR-2). The main AMP is cathelicidin, which is cleaved by kallikrein 5 (KLK-5) into the active peptide LL-37. This is the fundamental mediator for activating and controlling numerous processes: release of cytokines and metalloproteinases (MMP) by leukocytes, mast cells, and keratinocytes, regulation of the expression of extracellular matrix components, and increased proliferation of endothelial cells, causing angiogenesis. MMP-2 and MMP-9 are elevated on the skin of patients, exerting inflammatory, angiogenic, and dermal framework disruption functions in addition to helping in the activation of KLK-5, retrofeeding the system. MMP-9 is directly stimulated by the mite *Demodex folliculorum* (Df).^17^, ^19^, ^20^, ^21^, ^22^

The impairment of the cutaneous barrier in rosacea contributes to the pathophysiology of the disease, since its integrity is essential for the innate immune system. An increase in the loss of transepidermal water and a more alkaline pH is observed, possibly due to the activation of epidermal proteases, especially KLK-5. It is also important that Df can lead to the rupture of the skin barrier, causing micro abrasions on the skin, producing hypersensitivity in rosacea.^23^, ^24^, ^25^, ^26^, ^27^

In adaptive immunity, there is a predominance of cytokines of the Th1/Th17 pathways. IL-17 appears to induce angiogenesis through VEGF and affect the expression of LL-37 in keratinocytes.^28^, ^29^ Serum vitamin D levels in rosacea patients were higher than in the control group. This steroid hormone probably influences the pathway associated with TLR-2, KLK-5, and LL-37, altering the immune system.^17^, ^23^, ^24^

In neurovascular dysregulation, vanilloid and ankyrin receptors – present in neuronal tissues, in the endothelium, and in keratinocytes – can release important neuropeptides in the characteristic flushing of rosacea; they can be triggered by heat, cold, alcohol, spicy foods, and chemicals.^2^, ^7^, ^8^, ^11^, ^17^, ^30^, ^31^, ^32^

The pathophysiological mechanisms, in particular the inflammatory and vascular mechanisms, which act to potentiate the rosacea triggering and maintenance, induce the acceleration of proliferation and epidermal differentiation, as well as the dysfunction of the stratum corneum, decreasing the ability to attract and retain water, worsening the inflammatory process, and aggravating the damage to the skin barrier.^33^

### Triggering factors

One of the characteristics of rosacea is the possibility that signs and symptoms (especially flushing) are triggered by environmental factors or lifestyle habits. In 2002, the National Rosacea Society (NRS) conducted a study with 1,066 patients with rosacea to identify the main related factors; the most cited were the following: sun exposure (81%), emotional stress (79%), hot weather (75%), wind (57%), intense physical exercise (56%), alcohol consumption (52%), hot baths (51%), cold weather (46%), spicy foods (45%), humidity (44%), certain skin care products (41%), hot drinks (36%), certain cosmetics (27%), medicines (15%), and medical conditions (15%), among others.^34^

Despite not being mentioned in this research, later studies indicated the mite Df as an important triggering factor.^35^

In non-scientific surveys, also conducted by NRS, over 90% of the interviewed rosacea patients who identified and avoided their triggering factors presented improvement, in varying degrees.^36^

### Classification

In 2002, the NRS developed a classification that was revised in 2004, which provided standardized criteria for conducting research, analyzing results, and comparing data from different sources, serving as a diagnostic reference in clinical practice.^37^, ^38^ It classifies rosacea into four subtypes: (1) erythematotelangiectatic; (2) papulopustular; (3) phymatous; and (4) ocular. Although excellent from an educational standpoint, this classification clearly ignored the differences in the intensity of clinical manifestations, the possibility of progression between different forms, and even the chances of overlapping subtypes.^35^

The etiopathogenesis of rosacea was then unknown, and there were no histological or serological markers for the disease; therefore, the system was based merely on the characteristic morphology to provide a “framework” that could be updated in the face of new discoveries. Therefore, since its introduction, the NRS classification has been proposed as a provisional system, which should be modified/updated as scientific knowledge and clinical practice experience advance.^39^

Rosacea findings may encompass several subtypes, progress between different subtypes, demonstrate varying intensities, or even be pathognomonic (*e.g*., phyma).^40^, ^41^ A system based on phenotypes – observable characteristics that may result from genetic and/or environmental influences – provides the necessary means to evaluate and propose treatments individually, according to the presentation of each patient.^42^

The Rosacea Consensus (ROSCO) panel – composed of dermatologists and ophthalmologists from Africa, Asia (including India, China, and Singapore), Europe, North America, and South America – was the first to adopt and propose a global approach to address the diagnosis and classification of rosacea. The group aimed to establish an international consensus on diagnosis, severity, and treatment options, in order to improve diagnostic and therapeutic results.^42^ Soon thereafter, still in 2017, NRS, through a panel of experts, adopted criteria very similar to those proposed by the ROSCO panel, also recommending assessment by phenotypes.^39^

## Diagnosis and clinical aspects

### Diagnostic phenotypic criteria

Both the new ROSCO classification and the updated NRS consider two manifestations as signs or diagnostic criteria when present in isolation or associated with other manifestations (Table 1).^39^, ^42^

**Table 1:** Rosacea phenotypes. Diagnostic, major, and secondary characteristics.^a^

Diagnostic^b^	Major criteria^c^	Secondary criteria
Persistent centrofacial erythema associated with periodic intensification due to possible triggering factors	Flushing	Burning sensation on the skin
Phymatous changes	Papules and pustules	Ardency on the skin
	Telangiectasia	Edema
	Ocular manifestations:	Dryness of the skin
	• Telangiectasia on the margin of the eyelid	Ocular manifestations:
	• Blepharitis	• Accumulation of crusts and a collarette at the base of the eyelashes
	• Keratitis/Conjunctivitis/Scleroceratitis	
		• Irregularity of the eyelid margin
		• Tears evaporation dysfunction

a Adapted from Tan et al., 2017^39^ and Gallo et al., 2017.^42^

b These findings alone are sufficient for the diagnosis of rosacea.

c The presence of two or more findings is considered diagnostic.

#### Persistent centrofacial erythema

It presents a characteristic pattern that is intensified periodically by triggering factors. In patients with Fitzpatrick skin phototypes I to IV, this is the most frequent presentation of rosacea. Differential diagnosis with lupus erythematosus, seborrheic dermatitis, photodermatoses, and steroid-induced rosacea is important. In patients with phototypes V and VI, skin pigmentation can hinder clinical evaluation; however, irritation symptoms such as burning and stinging can help in the assessment. In these phototypes, papules and pustules may be the first clearly visible signs.^43^

#### Phymatous changes

In the newer classifications, this is a pathognomonic finding of rosacea.^39^, ^42^ It is more frequent in males and, although it can affect any area of the face, the nasal region is the most common (rhinophyma).^44^ It initially presents as a thickening of the skin, with erythema and edema, which is visibly inflammatory. It progresses with the proliferation of fibrous tissue and sebaceous glands, as well as accentuation of follicular orifices with sebum and keratin plugs that drain an unpleasant odorous material, a residual condition with no evident inflammation.^45^

### Major phenotypic criteria

The main phenotypic criteria of rosacea may be observed associated or unassociated with the diagnostic criteria. If they are not associated, at least two major criteria are required for diagnosis.^39^, ^42^

#### Flushing

It consists of diffuse erythema, which occurs in outbreaks of varying duration and frequency, intensifying centrofacial redness; long-term outbreaks are common. It often occurs in association with edema of varying degrees and can present with sensations of heat, burning, and/or pain. In individuals with high phototypes, it can be difficult to observe; at times, it is not observed, being only subjective. Its occurrence is linked to neurovascular stimuli triggered by several factors.^46^, ^47^

#### Papules and pustules

The papules are erythematous, arranged predominantly in the centrofacial region; some can be larger and deeper, and they may be accompanied by pustules. The association with erythema and edema of varying degrees is common. Comedones, when present, signal concomitance of acne unrelated to rosacea and assist in the differential diagnosis.^47^, ^48^, ^49^

#### Telangiectasias

Telangiectasias have a facial center location; as their presence in the nasal wings is frequent in the general population, they are not considered in the diagnosis. It is important to exclude steroid use and chronic actinic damage as causative factors. In patients with high phototypes, V and VI, dermoscopy can assist in the diagnosis.^39^

#### Ocular manifestations

They can be part of the primary or secondary criteria, and will be addressed in a separate topic.

### Secondary phenotypic criteria

In the ROSCO and NRS classifications, the secondary phenotypic dermatological manifestations are similar and include: burning sensation, stinging sensation, dryness, and edema. The difference between the two classifications lies only in ophthalmological criteria.^39^, ^42^

#### Ardency and burning sensations

Both are more frequent in patients with rosacea. Lonne-Rahm et al. evaluated patients with a burning complaint and demonstrated a higher incidence in this population, especially in the malar region.^50^ Pruritus, although it can occur, is not a characteristic symptom.

#### Edema

In rosacea, edema can be linked to vascular changes and inflammation, and its association with erythema and flushing is quite common. Although it is often associated with other manifestations, it can occur in isolation. It can be depressible or firm, self-limited or persistent, with variable duration. Both lymphatic or blood vessels can be implicated, and the dominance is variable.^46^, ^51^, ^52^

#### Dryness of the skin

The central region of the face can be rough and scaly, despite the patient reporting having “oily skin.” The coexistence of seborrheic dermatitis increases the difficulty of the diagnosis, as rosacea can simulate an eczematous condition.

#### Ocular rosacea

Ocular rosacea is regularly neglected by dermatologists, who tend to address only skin manifestations. Greater knowledge by dermatologists and ophthalmologists can improve the diagnosis and management of these patients.^52^ It is important to emphasize that this manifestation can behave independently of skin lesions, and may be present in mild, moderate, and severe degrees, or even in the absence of the main phenotypic criteria.^37^, ^39^

The ROSCO panel attributes the following ocular manifestations to an increased possibility of association with rosacea:^52^

• Telangiectasias of the margins of the eyelids: Vessels visible around the margins of the eyelids.
• Blepharitis: Inflammation of the eyelid margin, most commonly due to dysfunction of the Meibomian glands.
• Keratitis: Inflammation of the cornea that can lead to defects and, in the most severe cases, loss of vision.
• Conjunctivitis: Inflammation of the mucosa that lines the inner surface of the eyelids and bulbar conjunctiva. It is typically associated with vascular congestion and conjunctival edema.
• Anterior uveitis: Inflammation of the iris and/or ciliary body.

The new NRS classification also includes symptoms that commonly appear in rosacea, but are not specific. They include burning, stinging, photosensitivity, and foreign body sensation.^39^

In most cases, the diagnosis of ocular rosacea is eminently clinical. The assessment of telangiectasias in the margins of the eyelids and conjunctival interpalpebral erythemas, as well as the inspection of the meibomian glands and chalazion, can be performed without the aid of instruments and by non-specialized professionals. There are, however, conditions in which the aid of an ophthalmologist may be necessary, such as keratitis, scleritis, and infiltrates with corneal vascularization.^39^

## Methods

This consensus was elaborated by five dermatologists, from different Brazilian regions, experienced in rosacea, who were appointed by the Brazilian Society of Dermatology. An updated systematic review on the subject was carried out, based on scientific evidence, combined with personal experiences. Based on the modified DELPHI methodology, the experts contributed by indicating their agreement on a Likert scale, measured by the variables: (1) strongly agree, (2) partially agree, (3) neither agree nor disagree, (4) partially disagree, or (5) totally disagree. Thus, it was possible to identify the different levels of intensity of opinion on the same subject. All meetings were remote, and all members answered all questions. The data obtained were evaluated and the degree of agreement of the panel was greater than 90% for all items in the manuscript.

### Treatment

#### General measures

Rosacea is characterized by sensitive skin on the face, with compromised skin barrier and vascular hyper-reactivity. Many patients complain of exaggerated sensitivity to cleaning agents and cosmetics. Therefore, guidance on triggering and aggravating factors and general measures on skin care are essential in any form and severity for maintaining the integrity of the skin in the long term and for the success of the treatment.

The guidelines should be aimed at identifying and preventing exposure to triggering factors, which cause aggression to the skin barrier and/or vasodilation.^53^, ^54^, ^55^, ^56^, ^57^, ^58^, ^59^

General care must include gentle cleaning, only once or twice a day, with agents without soap, *i.e*., lotions for sensitive skin such as micellar water or with mild soaps, with pH 5.5; daily use of non-greasy moisturizers, similar in composition to the skin's natural moisturizing factor, preferably rich in ceramides, hyaluronic acid, glycerin, allantoin, licorice, niacinamide, plant oils (triglycerides, polyphenols, triterpenes, free fatty acids, phospholipids, antioxidants, such as tocopherol, *etc*.), and without alpha-hydroxy acids.^60^

As the inflammation compromises the barrier function, increasing the loss of transepidermal water, leaving the skin dry and sensitive, moisturizers with occlusive and humectant function should be applied, preferably at night, when the recovery of the epidermal barrier is slower, and permeability and water loss are higher. Moisturizers for sensitive skin are more suitable, some with green pigment, which are useful to mask erythema.^61^

Broad spectrum photoprotection should be used daily and continuously, with a protection factor above 30, preferably containing dimethicone, zinc oxide, or titanium dioxide to avoid irritating dermatitis, and be tinted, to act as color correction. Camouflage is very useful for transient or persistent erythema, reducing the impact on quality of life and the stress due to the appearance of the skin.

Even with care in choosing the products to be prescribed, there are always risks of adverse events, given the sensitivity of the skin; this should be informed to patients. The following products should be avoided: waterproof cosmetics, due to the difficulty of removal; tonics and astringents containing alcohol, menthol, camphor, or eucalyptus oil; and products with sodium lauryl sulfate, strong fragrances, fruit acids, and exfoliants.^54^, ^55^, ^56^, ^57^, ^58^, ^59^

#### Topical treatments

They aim to control flares, with therapeutic options that lead to effective control, good tolerability, satisfaction, and a positive impact on quality of life. It is relevant to emphasize that, although there is no cure, total improvement and long periods of remission are possible; in mild to moderate cases, topical treatments have a good level of confirmatory evidence and may be sufficient.

The most cited topical treatments in the literature are: 0.75% metronidazole in gel or cream and 1% in cream; azelaic acid 15% gel or 20% cream; α-1 adrenergic receptor agonists (brimonidine tartrate 0.5% gel and oxymetazoline 1% cream); and ivermectin 1% cream.^53^, ^54^, ^55^, ^56^, ^57^, ^59^ Although not yet commercialized in Brazil, 4% topical minocycline foam has also been described in the recent literature.^59^

In the 1990s, metronidazole was reported in studies; its main mechanisms of action are the anti-inflammatory effect and reduced Df density.^53^, ^54^ In Brazil, it is commercially available at 0.75% gel; other presentations are available in manipulation pharmacies.

Azelaic acid is considered effective for its anti-inflammatory activity established in *in vitro* studies, through the inhibition of neutrophil functions and the generation of reactive oxygen species (ROS).^62^, ^63^ A recent study has demonstrated its anti-inflammatory effect *in vivo* on adult women's acne, by significantly reducing the expression of TLR-2 which is also involved in the pathophysiology of rosacea.^64^ In rosacea, its effectiveness compared to the vehicle was demonstrated in a randomized study and confirmed in a systematic review.^65^, ^66^ In Brazil, azelaic acid is commercially available as 15% gel and 20% cream.

The α-1 adrenergic receptor agonists present in dermal vessels, particularly in arterioles, cause vasoconstriction. The use of 0.5% brimonidine gel was approved by the Food and Drug Administration (FDA) in 2014. There was a great expectation as a solution for the transient and persistent rosacea erythema; however, adverse events were reported in around 31% of patients.^67^ A study published in 2017 observed worsening of erythema, flushing, rebound, contact dermatitis, pruritus, and burning in about 10% to 20% of patients, with interruption of use by 17%. The authors suggested that its use could be helpful only in cases of persistent erythema^68^ In 2017, the FDA approved the use of oxymetazoline 1% cream, once a day in the morning for persistent erythema. Several authors have demonstrated its effectiveness in improving erythema by two degrees according to the scale used.^68^, ^69^ Adverse events such as dermatitis and/or erythema, paraesthesia, pain, dryness, and pruritus were observed in 8.2% of patients, most frequently in the first 90 days of treatment; only 3.2% of patients discontinued use, without worsening of the erythema. Rebound occurred in less than 1% of patients. Evidently, there was no effect on telangiectasias. The present authors consider that, due to the short time of use, it is not possible to ensure its safety, efficacy, and tolerability. In Brazil, only 0.5% brimonidine gel is approved, and already commercially available.

Ivermectin 1% cream was approved by the FDA in 2017, indicated for moderate to severe papulopustular rosacea, with less effectiveness in the milder form, with less lesions.^70^ Recently, its beneficial effects on ocular changes have been described.^71^ Its mechanism of action is based on inhibiting the cathelicidin pathway and reducing the density of Df, which activates toll-like receptor 4 (TLR-4), with the release of inflammatory mediators; it acts as an anti-inflammatory, with decreased expression of TNF-α and IL-1 beta.^70^ The role of Df in rosacea is still controversial; however, some authors suggest that rosacea-like demodecidosis and rosacea can be considered two phenotypes of the same disease that improve not only by the anti-inflammatory action of ivermectin, but also by its anti-parasitic action.^72^ Side effects were irritation, xerosis, pruritus, and itching.^73^ A systematic review published in 2018 confirmed the efficacy of topical ivermectin compared to other topicals, but no comparative studies with doxycycline and isotretinoin alone or in combination were retrieved.^74^ In Brazil, ivermectin for topical use is only avaiable in handling pharmacies.

Other topical agents for the treatment of rosacea, reported in older publications, include: 10% sodium sulfacetamide, 5% or 10% sulfur lotions, 5% benzoyl peroxide, and retinoids (0.05% retinaldehyde and 0.025% or 0.05% tretinoin). The last two present a high risk of causing irritative dermatitis and would only be justified if rosacea is associated with acne or photoaging.^53^, ^54^

#### Systemic treatments

More severe or refractory cases require isolated systemic treatment or, more commonly, association with the topic treatment.

The drugs for systemic use are antibiotics, especially from the group of cyclins (tetracycline, doxycycline, and minocycline), metronidazole, and isotretinoin, which will be detailed below, highlighting their adverse events that should be monitored. Although in clinical practice lymecycline is frequently used in Brazil, there is no evidence to support its use in the treatment of rosacea. Among the aforementioned oral medications, doxycycline and minocycline are available in manipulation pharmacies in Brazil, while the others are commercially available.^53^, ^54^, ^56^, ^57^, ^58^, ^59^

β-Adrenergic receptor antagonists, also called beta-blockers, such as carvedilol, atenolol, nadolol, and propranolol – for systemic use – have vasoconstrictive properties in the smooth muscle of dermal arterioles and do not act on capillaries. The use of carvedilol was published in a case report of severe and refractory rosacea, with high efficacy for erythema and few side effects; however, there is an increased risk of hypotension and bradycardia.^75^

#### Phenotype-based treatments

Global approach in the management of rosacea should be based on knowledge and choice of the various topical and systemic therapeutic options, according to the phenotype and severity of the disease.^53^, ^54^, ^56^, ^59^ At the end of this consensus, the experts’ treatment suggestions will be presented, based on phenotypic manifestations.

When transient erythema, also known as flushing, prevails, there is no treatment with established confirmatory evidence. General measures and skin care are advised. Soothing masks with chamomile, feverfew, green tea, *etc*. can be used. Topical alpha-adrenergic agonists and oral beta-blockers are no longer indicated.^53^, ^54^, ^58^, ^59^ The use of botulin in intradermal injections has been successfully suggested in case reports and may represent an alternative therapy that needs further studies.^76^, ^77^

Alpha-adrenergic agonists, such as 0.5% brimonidine tartrate in gel and 1% oxymetazoline in cream, are recommended for persistent erythema.^59^, ^67^, ^68^, ^69^, ^70^ Laser and intense pulsed light (IPL) can be used.

Telangiectasias should be treated with technologies such as IPL, with 550 or 600 nm filters and laser, with widely documented success.^78^ The laser mechanism of action is that of selective photothermolysis and the target chromophore is the hemoglobin pigment present in blood vessels.^79^ It is a safe and effective method, with secondary results, such as reduction of erythema, improvement of skin texture and, sometimes, reduction of papules and pustules. Multiple sessions (up to 15) are required at intervals of 1–3 weeks. The parameters must be adjusted individually, according to the phototype, severity, and tolerance, in order to minimize side effects. There are few reports in the literature about the time of remission of rosacea, but it is believed that it can reach 1 year.^78^, ^79^ Electrocauterization is an alternative.

For inflammatory papulopustular lesions, topical and/or systemic treatments are indicated.^53^, ^54^, ^55^, ^56^, ^57^, ^58^, ^59^ For mild to moderate forms, the order of preference includes azelaic acid, ivermectin, topical metronidazole, and doxycycline 40 mg/day; for severe forms, topical ivermectin, doxycycline 40 mg/day, and oral isotretinoin in a low daily dose (25–0.3 mg/kg), in off-label use.^58^ Other options, without confirmatory, are: tetracycline, lymecycline, minocycline, azithromycin, oral metronidazole, topical alpha-agonists, sulfacetamide, calcineurin inhibitors, and topical retinoids.

The anti-inflammatory action of doxycycline was well documented in a review article published in 2007.^80^ The 40 mg/day dose combined with the use of 1% metronidazole gel was effective and safe.^81^ The dose of 40 mg was compared to that of 100 mg/day, with the same efficacy.^82^ Likewise, effectiveness has been demonstrated with the combined use of 1% ivermectin cream.^83^ In Brazil, it is not commercially available, and only manipulation pharmacies offer it. However, it is important to note that it is different from the one available in the United States, which has slow release, a factor that may cause some change in bioavailability and pharmacokinetics and consequently on the efficiency of the drug.

Oral isotretinoin is only approved for moderate to severe acne, providing healing or prolonged remission. Many off-label indications, such as rosacea, have been reported.^84^ The perspective of efficacy is related to the breadth of its mechanisms of action, particularly the potent inhibition of sebaceous glands and the anti-inflammatory properties by TLR-2 modulation, with increased expression in the keratinocytes in rosacea.^85^ Isotretinoin has a high degree of recommendation for moderate to severe pustular rosacea, or when recurrent or unresponsive to antibiotic therapy. In 1994, a Chilean author reported a series of cases on the use of isotretinoin in severe rosacea, for periods of 3–6 months, with rapid remission of papules and pustules, improvement of ocular manifestations, few side effects, and maintenance of results for an average 14.8 months.^86^ In 2010, a German multicenter, double-blinded, randomized study including 573 patients with papulopustular and phymatous rosacea compared different doses of isotretinoin (0.3, 0.5, 1 mg/kg/day) with doxycycline 100 mg/day for 14 days and then 50 mg/day and placebo. It was observed that isotretinoin 0.3 mg/kg/day was more effective than placebo, and was equally or even more efficient than doxycycline, with fewer side effects than in higher doses.^87^ To better control relapses, studies have suggested a treatment schedule with isotretinoin 10–20 mg/day for 4–6 months, followed by maintenance with continuous microdoses (0.03–0.17 mg/kg/day – mean: 0.07 mg/kg/day) for up to 33 months, resulting in a better option than multiple cycles of antibiotic therapy.^88^ Another study used intermediate doses, *i.e*., an initial daily dose of 20 mg/day for 4 months, with rapid reduction of erythema and inflammatory lesions and subsequent slow and progressive dose reduction, for 6 months, up to 20 mg/week, with recurrence rate of 45% at the 11-month follow-up.^89^ Oral isotretinoin, associated with oral corticosteroids (prednisone, 40–60 mg/day) is considered the treatment of choice for rosacea fulminans, a very severe variant. It is recommended to start with a low daily dose, 0.2–0.5 mg/kg, and then increase to 0.5–1 mg/kg for 3–4 months.^90^

Side effects are well known, dose-dependent, predictable, preventable and controllable with early care. The most common are mucocutaneous, such as: cheilitis (present in 100% of patients), xerophthalmia, conjunctivitis, nasal dryness, epistaxis, and irritant dermatitis.^91^ Laboratory changes such as elevated liver enzymes, triglyceride, and cholesterol levels, elevated LDL fraction, and decreased HDL fraction may occur.^92^, ^93^ The association with depression, suicidal ideation, or attempted suicide, as well as the onset of inflammatory bowel disease related to the use of oral isotretinoin was not demonstrated in the literature.^94^, ^95^, ^96^, ^97^ Clinical and laboratory assessment should be made before and repeated 8 weeks after the start of treatment; new assessments are required only for altered parameters.^93^, ^98^ Special attention should be paid to teratogenicity, since rosacea often affects adult women of childbearing age.^99^, ^100^

In phymatous rosacea, there is hyperplasia of sebaceous glands, connective tissue, and blood vessels. By suppressing the activity of the sebaceous gland, oral isotretinoin may delay progression when used in the pre-fibrotic or inflammatory phase. Some reports demonstrated a reduction in size, number, and activity of the sebaceous glands; the dose of 1 mg/kg/day, for 18 weeks, reduced the nasal volume from 9% to 23%. Better results are obtained in young patients, but there is recurrence after discontinuation of the drug.^101^ A case report demonstrated the efficacy of isotretinoin 20 mg/day, for 6 months, with recurrence after suspension and maintenance of the result with 10 mg/day for a prolonged period.^102^ Other alternatives are doxycycline 100 mg/day and intralesional infiltration with corticosteroids. Fibrotic or non-inflammatory phymas are treated with surgery, ablative laser, electrosurgery, dermabrasion, and radiofrequency, which will be addressed later.^58^

The treatment of ocular rosacea should include eyelid hygiene, use of artificial tears for eye lubrication, and sunglasses; in mild cases, 0.75% metronidazole; ivermectin, fusidic acid applied to the eyelids, 0.05% cyclosporine in eye drops or emulsion, topical corticosteroids; in severe cases, oral doxycycline.^54^, ^55^, ^58^, ^103^ Few studies with isotretinoin were retrieved in the literature, but the tendency is for the drug to improve signs such as blepharitis and conjunctivitis; low daily doses of 10 mg/day are recommended.^54^ Topical ivermectin, of recent use, has shown satisfactory results in ocular changes.^71^ Other options mentioned in the literature are omega 3, erythromycin, azithromycin, and oral corticosteroids. Topical maintenance treatment and evaluation by an ophthalmologist are recommended.

Granulomatous rosacea is rare; therapeutic options can include isotretinoin, 0.7 mg/kg/day for 6 months, cyclins, dapsone, laser, photodynamic therapy, brimonidine, azelaic acid, topical metronidazole, benzoyl peroxide, and topical and systemic corticosteroids.^104^

#### Maintenance treatment

The recommendation is to use topical medication, such as azelaic acid, metronidazole, and ivermectin. In selected cases, isotretinoin in microdoses (20 mg/week) can be used, with strict laboratory and risk-of-pregnancy control.

#### Technology and surgery

Various sources of laser, IPL, and light-emitting devices can be used in the treatment of rosacea. Radiofrequency, ultrasound, electrosurgery, and microneedling can also be indicated in certain cases.^42^, ^76^, ^105^, ^106^, ^107^, ^108^, ^109^, ^110^, ^111^, ^112^, ^113^, ^114^, ^115^, ^116^, ^117^, ^118^, ^119^, ^120^, ^121^, ^122^, ^123^, ^124^, ^125^, ^126^, ^127^, ^128^

The main applications of technological equipments are for the improvement of erythema, telangiectasias, and phymas.^106^, ^107^

Three principles are fundamental in the handling of lasers and IPL: choosing the appropriate wavelength for the target chromophore, choosing a pulse duration shorter than the thermal relaxation of this chromophore, and applying enough energy to destroy the target within an appropriate time interval. New equipments present robust cooling capacity, high energies in short pulses, and larger tips. These elements represent effectiveness, speed, and security. Overlapping pulses and making multiple passes can also increase effectiveness.^106^, ^129^

IPL: It is well indicated in the treatment of telangiectasias. It may act on erythema, mainly perilesional, and on papules and pustules, depending on the cutting filter. Inflammatory symptoms, such as pruritus, edema, burning sensation, and pain may be alleviated.^59^, ^106^, ^107^, ^111^, ^122^, ^130^, ^131^, ^132^, ^133^, ^134^, ^135^, ^136^, ^137^

The target chromophore is hemoglobin, whether oxyhemoglobin, present in red colored lesions, deoxygenated hemoglobin, present in bluish lesions, or methemoglobin. The mechanism of action is based on photothermolysis or thermal damage to the vessels, which induces intravascular coagulation.^114^, ^123^, ^138^, ^139^, ^140^

IPL works by collating vessels, remodeling collagen, and reorganizing connective tissue, actions that provide longevity of the effects induced by this technology.^139^, ^141^

The lesions are treated with one or two pulses, until the start of vasospasm, associated with mild erythema and/or edema. Vessel rupture is not desired, as it leads to hemosiderin deposition and possible skin hyperpigmentation. Telangiectasias located on the nasal wings are more resistant and prone to relapse.^122^, ^130^

The use of filters according to the phototype is suggested. If vasoconstriction is not noticed, energy should be increased and then, if necessary, the pulse duration should be reduced. In general, reducing the spot requires increased energy (greater fluence) and vice versa. Longer wavelengths are effective for treating deeper vessels, while shorter ones target more superficial vessels. Multiple sessions (at least three) are required, at intervals of 1–3 weeks.^110^, ^142^

Pulsed dye laser (PDL)(585 nm or 595 nm): well indicated in erythema and telangiectasis. The introduction of longer pulses (between 20–40 ms) reduced the occurrence of purpura and hyperpigmentation, maintaining the effectiveness.^110^, ^111^, ^133^, ^137^, ^143^, ^144^, ^145^

ND: Long pulse YAG (1.064 nm): good efficacy in the treatment of facial telangiectasia. In deeper vessels and in telangiectasias larger than 1 mm, it was superior to IPL. Studies report some effectiveness in the treatment of papules and pustules.^106^, ^111^, ^120^, ^133^, ^146^, ^147^, ^148^, ^149^

KTP – potassium-titanyl-phosphate (532 nm): more effective in small and superficial telangiectasias. Its use is limited in the higher phototypes due to the risk of hyperpigmentation.^111^, ^143^, ^150^, ^151^, ^152^, ^153^, ^154^, ^155^

Alexandrite laser (755 nm): less frequently used in rosacea, but has potential in subdermal telangiectasias.^111^, ^121^

Pro-yellow laser: 577 nm laser emitting 100% yellow light energy. A study in Turkey reported the effectiveness of its use in the treatment of erythema and facial telangiectasias in rosacea.^156^

Light emitting diode (LED): its action comes from low intensity non-thermal irradiation, with modification of cellular activity and anti-inflammatory effect. It has an additional indication in erythema and inflammatory lesions.^111^, ^133^, ^157^, ^158^, ^159^, ^160^, ^161^, ^162^, ^163^, ^164^, ^165^, ^166^

Radio frequency: it is part of the electromagnetic spectrum and provides energy in the form of electricity. Its effectiveness in the treatment of facial erythema, papulopustular lesions, and rhinophyma is variable.^113^, ^115^, ^167^, ^168^

Ultrasound: the device used in dermatology is similar to that used for imaging, but its energy is highly convergent and has higher frequencies. In rosacea, there are reports of improvement of erythema and telangiectasias.^169^, ^170^

Electrosurgery: it can be used in low configurations to treat telangiectasis, but there is a risk of thermal damage, with punctate or linear scars.^115^

Microneedling: with or without transdermal delivery of active agents to the skin through microchannels, can improve erythema and telangiectasia in rosacea. The lesion should be mild to moderate. The most frequently mentioned active agents are botulin and tranexamic acid, which acts to restore skin permeability and suppress angiogenesis.^125^, ^126^, ^127^, ^128^, ^171^, ^172^, ^173^, ^174^

Photodynamic therapy (PDT): studies with multiple sources of light and laser for activation of topical aminolevulinic acid in PDT for the treatment of rosacea have resulted in divergent responses, from insignificant to effective.^175^, ^176^, ^177^, ^178^

#### Phyma treatment

Non-inflammatory phyma: they represent a therapeutic challenge and no method is universally adopted as the gold standard.^42^, ^106^, ^107^, ^153^, ^153^, ^171^, ^179^, ^180^, ^181^, ^182^, ^183^, ^184^, ^185^, ^186^, ^187^, ^188^

Techniques that cause less damage to adnexal structures, along with minimal surgery time and costs, are preferred. Each method presents varying degrees of risk, related to low hemostasis, insufficient tissue removal, and scarring. Treatments can be excisional or ablative.^182^, ^183^

#### Excision

The techniques involve conventional surgery, removing the phyma through shaving, scissors, scalpel blade, and/or electrocoagulation, electrosurgery with wire in loop, radiofrequency, and laser ablation.

Surgical excision is the basis of treatment for rhinophyma. It can be divided into four main stages: delamination/decortication of the excess phymatous tissue, refinement of the nasal contour, hemostasis, and post-operative care.^42^, ^106^, ^182^, ^183^, ^184^, ^185^ There are numerous reports of the use of dermabrasion to refine the removal of phymatous tissue. The main risks are intraoperative bleeding, unsightly scars, and excessive tissue removal.^42^, ^106^, ^182^, ^183^, ^189^, ^190^, ^191^, ^192^

#### Ablatives

CO_2_ laser (10,640 nm): used in conventional ablative mode, or fractionated in milder phymas, with more sessions for satisfactory results.^42^, ^106^, ^133^, ^182^ Despite the risk of permanent depigmentation, texture changes, and scarring, cosmetic results are usually good.^111^, ^114^, ^143^, ^182^, ^193^, ^194^, ^195^

Er-Yag (erbium: yttrium-aluminum-garnet) laser (2,940 nm): similar to the CO_2_ laser, induces high temperatures in the target, resulting in vaporization and ablative phyma correction.^42^, ^114^

Dermabrasion: isolated or in association with other methods, promotes excellent response.^196^, ^197^, ^198^, ^199^, ^200^, ^201^, ^202^

Trichloroacetic acid (30%, 50%, 70%, or 90%): isolated or in association, usually with dermabrasion and/or tangential exeresis, provides favorable results.^179^, ^183^, ^189^, ^203^, ^204^

Cryosurgery with liquid nitrogen: acts through direct freezing action and by formation of vascular thrombi.^202^

#### Botulinum toxin

It has recently been used in the treatment of erythema, flushing, and inflammatory lesions of rosacea, especially when other therapies are ineffective. Its application is intradermal to prevent muscle dysfunction. It should be used in larger dilutions and, to date, its mechanism of action is still controversial.^76^, ^112^, ^133^, ^171^, ^205^, ^206^, ^207^, ^208^, ^209^, ^210^, ^211^, ^212^

It is postulated that it inhibits the release of neuropeptides associated with vasodilation and inflammation, such as acetylcholine and vasoactive intestinal peptide, or that the toxin prevents the release of neuropeptides involved in sebaceous activity, vascular homeostasis, and inflammation, such as substance P, calcitonin, and glutamate.^120^, ^178^

The therapeutic benefits may result from the blocking effects of acetylcholine on the erector muscles of the hair and on the muscarinic receptors of the sebaceous glands. The improvement reported in erythema, flushing, and inflammation results from the interruption of the release of acetylcholine in the peripheral autonomic nerves of the cutaneous vasodilator system, together with the inhibition of the release of inflammatory mediators.^120^, ^213^

In 2004, in a case report, after PDL failure for persistent erythema, a group applied botulin type A (BTX-A) to the centrofacial region, diluting 100 U in 5 mL of 0.9% saline solution in a proportion of 2 U per 0.1 mL, in 1 cm intervals, adding 10 U (0.5 mL) in each hemiface. The outcome was improvement in symptoms.^209^

In 2012, Dayan et al. performed a study injecting onabotulinum toxin in 13 patients with rosacea, diluting 100 U in 7 mL of 0.9% saline solution, resulting in 1.4 U for each 0.1 mL. At each injection site, 0.05 mL of the product was applied, spaced 0.5 cm apart, adding 8–12 U per hemiface. After 1 week, a reduction in transient erythema, persistent erythema, and inflammation were observed; the results persisted for 3 months.^112^, ^211^

Another report termed the combination of BTX-A microinjection and associated IPL in the treatment of rosacea as mesobotox, with good response. The vial was reconstituted with 10 mL of 0.9% saline solution. Each hemiface received eight applications of 0.1 mL of BTX-A (1 U of BTX-A per application point, for a total dose of 8 U) 2 cm apart. The forehead was also inoculated with five applications of 0.1 mL.^214^

Abobotulinum toxin A and incobotulinum toxin A have demonstrated good results in studies of rosacea treatment.^205^, ^206^, ^215^, ^216^

In Brazil, in 2018, a study demonstrated improvement of transient and persistent erythema for approximately 6 months with BTX-A in a dilution of 100 U to 5 mL of 0.9% saline solution, 0.2–0.5 U per application point. In that study, intradermal injections were applied to the malar regions, with an interval of 0.5 cm per point of application, totaling a volume that varied from 6 to 15 U per affected malar region (12–30 total units, equivalent to 0.6 to 1.5 mL of the dilution).^213^ This dilution and application method is recommended by the authors of this consensus.

Similar efficacy was observed in another Brazilian study that used onabotulinum toxin A in rosacea patients with erythema and inflammatory lesions, with good response.^217^

Other authors failed to reproduce the benefits of BTX-A in facial erythema. These divergent results may simply be a reflection of different pathophysiological mechanisms and various rosacea subtypes.^207^, ^218^

### Quality of life

As rosacea affects visually apparent areas of the skin, it usually has a strong impact on patients’ quality of life, which is often underestimated by dermatologists. Studies demonstrate that the disease can lead to depression, anxiety, shame, low self-esteem, and social phobia.^219^, ^220^, ^221^

Stress can be a triggering factor, and is related to vascular dysregulation and skin immunity resulting from the release of neuropeptides after excessive nerve stimulation. Therefore, failure to control symptoms can aggravate stress, resulting in a vicious cycle.^219^, ^220^, ^221^

RosaQol is a specific quality of life assessment instrument for rosacea, consisting of a questionnaire with 21 questions. Some studies suggest that RosaQol presents high reliability and validity, in addition to high consistency with the internationally accepted DLQI; however, other authors do not corroborate this statement.^52^, ^222^ RosalQol has already been translated and validated for Brazilian Portuguese, and should be considered as a measure of effectiveness in future clinical studies.^223^

### Comorbidities

Once considered a limited skin disorder, rosacea has been described in association with systemic diseases.^224^, ^225^, ^226^

In 2015, Hua et al. published an important study, describing rosacea as a “systemic inflammatory disease,” showing similarities with psoriasis in relation to the risk of cardiovascular (CV) disease, possibly because both diseases have altered innate immunity, increased cathelicidin and C-reactive protein, in addition to decreased paraoxonase activity, which are predictors of CV events. They concluded that patients with rosacea should be alerted to possible CV events, especially in relation to arterial hypertension, dyslipidemia, and coronary artery disease.^226^

More recently, in addition to CV diseases, other disorders have been described as associated with rosacea: neurodegenerative diseases (Parkinson's disease, multiple sclerosis, Alzheimer), neurological disorders (migraine, glioma), psychiatric disorders (depression, anxiety, obsessive-compulsive disorder, social phobia, stress), intestinal disease (Crohn's disease, ulcerative colitis, celiac disease, dysbiosis, *H. pylori* infection, small intestinal bacterial overgrowth), oncological diseases (thyroid, breast and liver cancer, non-melanoma skin tumors), and autoimmune diseases (diabetes mellitus, rheumatoid arthritis, multiple sclerosis, frontal fibrosing alopecia), among others.^224^, ^224^, ^225^, ^226^, ^227^, ^228^, ^229^, ^230^, ^231^, ^232^, ^233^, ^234^, ^235^, ^236^, ^237^, ^238^, ^239^, ^240^, ^241^, ^242^

In an attempt to explain the coexistence of these systemic diseases with rosacea, studies have been designed to search for similar elements, such as the sharing of genetic and environmental factors, or of immunological and cellular characteristics. However, further research is needed to consolidate these associations.^224^, ^225^, ^229^, ^243^, ^244^

Physicians should be aware of the possibility of comorbidities, and should be alert and vigilant, since there is a possibility that rosacea is only the tip of the iceberg of the patient's systemic impairment.

### Final considerations

Because it is a chronic inflammatory disease, with different clinical manifestations and with no possibility of cure at the present moment, the therapeutic management of rosacea is highly challenging.

This Brazilian consensus, prepared by five specialists from the Brazilian Society of Dermatology, aims to guide the dermatologist in the therapeutic approach of rosacea, also providing updated information on its epidemiology, pathophysiology, clinical manifestations, diagnostic methods, and comorbidities. These data converge in the quest to improve patients’ quality of life.

Several consensuses, as well as a Cochrane Library systematic review, published in 2015 and updated in 2019, were retrieved in the recent literature on rosacea treatment. The systematic review published in 2015 included 106 controlled and randomized studies with 13,631 patients, and the 2019 update included 46 more studies, totaling 152, and 20,944 patients. The conclusions regarding the levels of evidence were: (1) high for brimonidine and moderate for oxymetazoline for temporary reduction of persistent erythema; (2) low to moderate for laser and IPL for erythema and telangiectasias; (3) high for azelaic acid and ivermectin, moderate to high for doxycycline 40 mg and isotretinoin, moderate for topical metronidazole, topical and oral minocycline (same as doxycycline 40 mg), and low for tetracycline and minocycline in low doses for papules and pustules; (4) moderate for oral omega-3 and low for ophthalmic cyclosporine in emulsion and doxycycline for ocular rosacea; (5) association of topical and systemic treatments is useful; and (6) maintenance treatment is recommended.^245^, ^246^, ^247^, ^248^

Based on personal experiences, associated with literature reviews, the authors of this consensus prepared a table on the recommendations of treatment options to be used in each clinical manifestation of rosacea. These recommendations are summarized in Table 2.

**Table 2:** Recommendations of Brazilian experts on therapeutic options for rosacea, according to clinical manifestations.

Erythema		Inflammatory lesions		Telangiectasias	Phymas		Ocular rosacea	Granulomatous rosacea
Transient	Persistent	Mild/moderate	Severe		Inflammatory	Non-inflammatory		
Soothing masks with chamomile, feverfew, green tea etc.	Topical alpha-adrenergic agonists (brimonidine)	Azelaic acid	Topical ivermectin	Electrocauterization or other technologies (laser or pulsed light)	Oral doxycycline	Surgery	Lubricating eye drops	Oral isotretinoin (0.7 mg/kg/day for 6 months)
		Topical ivermectin				Laser		
		Topical metronidazole	Oral doxycycline (40 mg/day)		Oral isotretinoin	Dermabrasion	Cyclosporine emulsion	Oral dapsone
	Technologies (pulsed light or laser)							Tetracycline
		Doxycycline 40 mg/day	Oral isotretinoin (0.25–0.3 mg/kg/day)		Intralesional corticosteroids	Trichloroacetic acid	Metronidazole or ivermectin on the eyelids	Pulsed-dye laser
								Photodynamic therapy
Intradermal botulinum toxin in large dilutions (as cited in the text)	Intradermal botulin in large dilutions (as cited in the text)	Other options: tetracycline, azithromycin, limecycline, minocycline, topical alpha-agonists, sulfacetamide, calcineurin inhibitors, topical retinoids						Topical metronidazole
							Doxycycline 40 mg/day Erythromycin Azithromycin Oral corticosteroids Oral omega 3	
General measures: avoid triggering factors; clean skin with soap-free agents or mild soaps with pH 5.5, photoprotection; use non-greasy moisturizers, with a composition similar to the skin's natural moisturizing factor; use cosmeceuticals (chamomile, green tea, niacinamide, feverfew, licorice).								

## Financial support

None declared.

## Authors’ contributions

Clivia Maria Moraes de Oliveira: Statistical analysis; approval of the final version of the manuscript; design and planning of the study; drafting and editing of the manuscript; collection, analysis, and interpretation of data; effective participation in research orientation; critical review of the literature; critical review of the manuscript.

Luiz Mauricio Costa Almeida: Approval of the final version of the manuscript; drafting and editing of the manuscript; critical review of the literature; critical review of the manuscript.

Renan Rangel Bonamigo: Approval of the final version of the manuscript; drafting and editing of the manuscript; critical review of the literature; critical review of the manuscript.

Carla Wanderley Gayoso de Lima: Approval of the final version of the manuscript; drafting and editing of the manuscript; critical review of the literature; critical review of the manuscript.

Ediléia Bagatin: Approval of the final version of the manuscript; design and planning of the study; drafting and editing of the manuscript; critical review of the literature; critical review of the manuscript.

## Conflicts of interest

The members of this consensus declare that they participated in scientific meetings, lectures and/or received support for events from the following pharmaceutical laboratories: Clivia Maria Moraes de Oliveira Carneiro (Janssen, Novartis, Sanofi), Luiz Mauricio C Almeida (Galderma, Leo Pharma), Ediléia Bagatin (Leo Pharma, USK, Natura, Galderma, Pierre-Fabre Dermo Cosmétique, Gelita, Douglas Pharmaceuticals). Renan Rangel Bonamigo and Carla Wanderley Gayoso de Lima declare no conflicts of interest.
